# Ultra-fast kinematic vortices in mesoscopic superconductors: the effect of the self-field

**DOI:** 10.1038/s41598-020-75748-5

**Published:** 2020-10-29

**Authors:** Leonardo Rodrigues Cadorim, Alexssandre de Oliveira Junior, Edson Sardella

**Affiliations:** 1 Departamento de Física, Faculdade de Ciências, Universidade Estadual Paulista (UNESP), Caixa Postal 473, Bauru, SP 17033-360 Brazil; 2 Instituto de Física Gleb Wataghin, Universidade Estadual de Campinas, P.O. Box 6165, Campinas, Sao Paulo CEP 13083-970 Brazil

**Keywords:** Condensed-matter physics, Superconducting properties and materials

## Abstract

Within the framework of the generalized time-dependent Ginzburg–Landau equations, we studied the influence of the magnetic self-field induced by the currents inside a superconducting sample driven by an applied transport current. The numerical simulations of the resistive state of the system show that neither material inhomogeneity nor a normal contact smaller than the sample width are required to produce an inhomogeneous current distribution inside the sample, which leads to the emergence of a kinematic vortex–antivortex pair (vortex street) solution. Further, we discuss the behaviors of the kinematic vortex velocity, the annihilation rates of the supercurrent, and the superconducting order parameters alongside the vortex street solution. We prove that these two latter points explain the characteristics of the resistive state of the system. They are the fundamental basis to describe the peak of the current–resistance characteristic curve and the location where the vortex–antivortex pair is formed.

## Introduction

The Ginzburg–Landau theory of superconductivity states that, in the presence of an applied current, a superconducting sample can sustain homogeneous superconductivity until the current reaches a critical value. In specific, this refers to the Ginzburg–Landau pair-breaking current density, $J^{GL}_c$, where the sample transitions to the normal state. Moreover, phase-slip phenomena enable superconductivity not to be destroyed at currents greater than $J^{GL}_c$. These coexist with a voltage difference across the sample in a resistive state.

This mechanism occurs in both thin filaments and wide superconducting film samples. Thin filaments possess dimensions perpendicular to the current flow that are much smaller than the Ginzburg–Landau coherence length, $\xi$. The phase-slip occurs at the Phase-Slip Centre (PSC), where the superconducting order parameter, $\psi$, periodically reaches zero magnitude with a phase drop of $2\pi$^1^. Wide films possess only one dimension that is less than the coherence length, $\xi$. The resistive state can be realized using two different processes: a Phase-Slip Line (PSL) and a vortex street. A PSL is analogous to the PSC for two dimensions, where the order parameter and the phase drop occur at a line perpendicular to the current flow in the sample. A vortex street, however, is a state where kinematic vortices move along a line perpendicular to the applied current of suppressed superconductivity^2^. Although the order parameter is very small along this vortex street, its phase carries two singularities where $\psi$ is consistently zero. They have been experimentally observed by Sivakov et al.^3^, who measured them using the Shapiro steps under microwave radiation produced by annihilating the kinematic vortex–antivortex (V–Av) pairs. In addition, kinematic vortices posses different characteristics from both Abrikosov and Josephson vortices. In particular, their velocities, as investigated theoretically and experimentally by Jelić et. al.^4^ and Embon et. al.^5^, can be greater than Abrikosov vortices and smaller than Josephson vortices.

A number of numerical works address resistive states in wide superconducting films. Andronov et al.^6^ simulated homogeneous and inhomogeneous wide superconducting films and encountered both PSL and vortex street solutions. In addition, Weber and Kramer^2^ investigated a similar configuration and provided solutions to a larger set of initial conditions and sample parameters. Berdiyorov et al.^7^ followed the changing states of a superconducting film while increasing the applied current. They studied the *I–V* characteristics of the sample, as well as the velocity and nucleation/annihilation position of the pair of kinematic vortices. They also investigated the influence of a perpendicular applied magnetic field on these physical quantities. He et. al.^8,9^ considered the effects of narrow slits inside the superconducting film and followed the behavioral changes of both kinematic vortices and PSLs on such systems. By varying the size and angle of the narrow slits, they encountered several different configurations when increasing the applied current. In a different system, Xue et al.^10^ studied the effects of radially injected currents on a square superconducting film containing a square slit at its center. They found that the current caused the kinematic vortices to rotate around the square, inducing a voltage oscillation. The increased external current motion of the vortices depended on the magnitude of the applied field. When investigating a finite superconducting stripe, Berdiyorov et. al.^11^ found that an increase in the $\gamma$ parameter, which is proportional to inelastic collision time, caused the phase slip process to occur in a larger current range. In addition, for large values of $\gamma$, a small applied magnetic field increased the critical current at which the system transits to the normal state. Moreover, heat dissipation on the resistive state contributed to quantitative changes in the size of voltage jumps and the value of the critical currents. However, it did not lead to any new qualitative features^12,13^. Lastly, Barba-Ortega et al.^14^ investigated the influence of a sample’s rugosity and found that it influenced both the critical currents and the kinematic vortex velocity.

To the best of our knowledge, none of the works in the present literature have considered the effects of the magnetic self-field induced by the internal currents in the superconducting sample to study the behavior of ultra-fast kinematic vortices. The reader should note, though, that this have been done for slowly moving Abrikosov vortices^15^. The aim of this paper is to show that, although small, the effects of the self-field are not negligible and produce important consequences to the resistive state, specifically the dynamic of the kinematic V–Av nucleation and annihilation, and the peaks present in the resistive characteristic curve.

## Results and discussion

**Figure 1:**
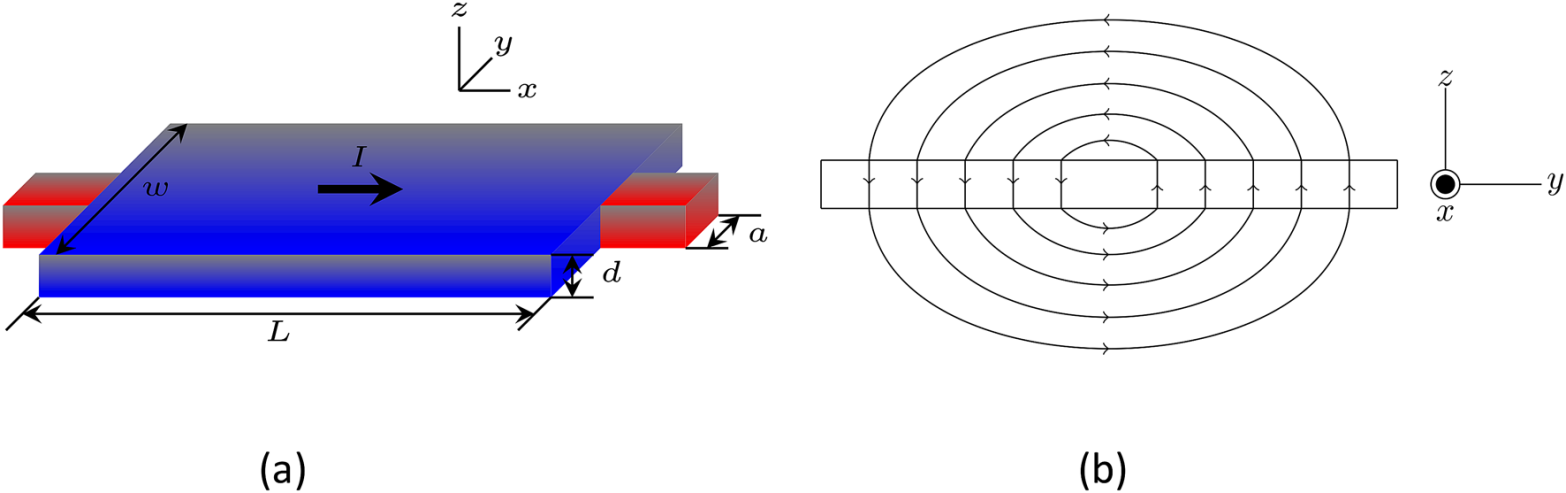
(**a**) A schematic view of a mesoscopic superconducting stripe. The metallic contacts are attached to both sides of the film, through which an external applied transport current, *I*, is injected. The dimensions are indicated in the figure. (**b**) According to the Ampère law, the transport current yields a self-field with streamlines as illustrated. The current flows in the *x* direction along the film.

We investigated a system consisting of a stripe attached to two metallic contacts on both sides, through which an applied current density, $J_a$, was injected. The length and width of the stripe are denoted by *L* and *w*, respectively. The width of the normal contacts is represented by *a*. The thickness is represented by $d\ll \xi ,\lambda$, while $\lambda$ represents the field penetration depth. Figure 1b illustrates the local magnetic self-field produced by the applied current. The local magnetic field was assumed to be perpendicular to the stripe. The validity of this approximation is discussed in more detail in “Methods” section and in the supplementary material.

We considered mesoscopic superconducting stripes of dimensions $12\xi \times 8\xi$. We assumed that the size, *a*, of the normal contact responsible for the injection of current in the superconducting stripe was equal to the sample width, $a = 8\xi$. The Ginzburg–Landau parameter, $\kappa$, and the constant, $\gamma$, were assumed to be 5.0 and 20, respectively. Note that, given the thin film geometry, this is an effective $\kappa$ value, which depends on the thickness of the stripe^16^. In most cases, the results were displayed as $I=aJ_a$ (the total current injected per unit length) rather than $J_a$. In the numerical simulations, we adiabatically increased the transport current in steps of $\Delta I = 0.079 I_0$ until the whole sample reached the normal state. In all calculations, the external magnetic field was $H=0$; here $I_0=\xi J_0$ (see “Methods” section for the definition of $J_0$, just after Eq. (6)). The boundary conditions for the local magnetic field did not account for the external applied field since we are interested exclusively in the effect of the the self-field (see “Methods” section for details of the theoretical formalism used).

**Figure 2:**
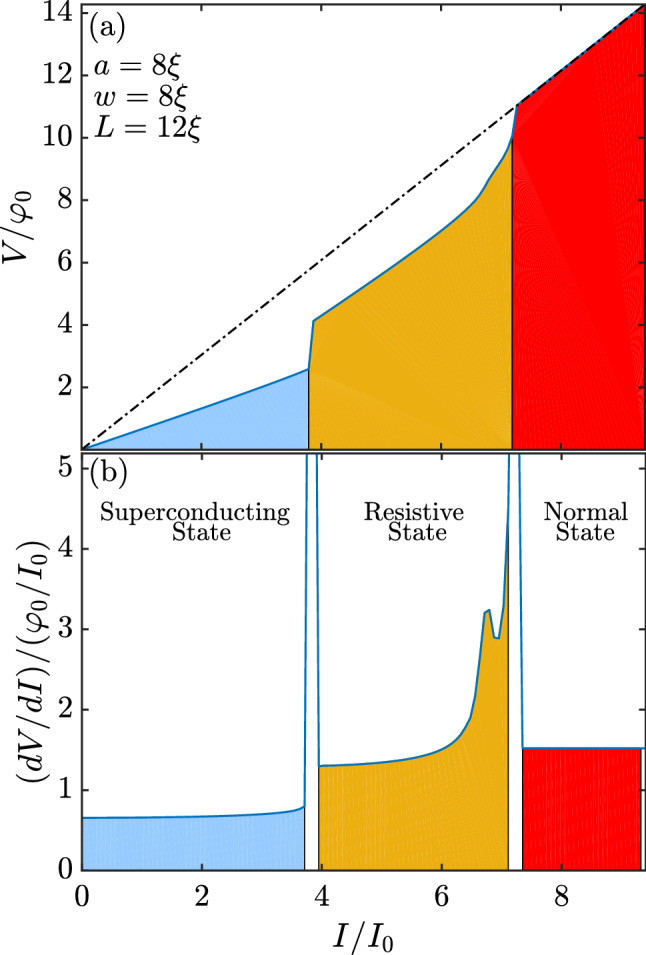
(**a**) The *I–V* characteristics of the system with a normal contact of size $a=8 \xi$. (**b**) The differential resistance for the same system. The blue, yellow, and red regions represent the Meissner, resistive, and normal states, respectively.

In Fig. 2a we show the current-voltage characteristics for the system. For currents where $I < 3.792 I_0$, the superconducting sample was in the Meissner state, without any dissipation process: the finite voltage presented is caused solely by the normal contacts. At $I = 3.792 I_0$, a voltage step occurred in the *I–V* characteristics and the system went into a resistive state. This resulted in the formation of a vortex street with suppressed superconductivity at the center of the sample and perpendicular to the applied current, where a pair of kinematic V–Avs moved from the edges towards the center of the sample. This is the first manifestation of the effects of the self-field, since when $a=w$, simulations neglecting the self-field reported in the literature^2,7^ showed that the vortex street did not occur. Instead, the resistive state found in these cases is characterized by a time periodic formation of PSLs at the center of the sample. This remarkable difference to the results of investigations which disregarded the self-field shows the importance of such effect to the resistive state.

The currents induced by the self-field are responsible for enhancing the inhomogeneity of the supercurrent distribution along the width of the sample, which are otherwise approximately uniform^7^. This break of homogeneity was responsible for the formation of a PSL for the initial parameters. It favors a solution that is now dependent on the *y* coordinate: the vortex street solution. This does not prohibit the existence of the PSL solution for smaller samples where the current distribution is more homogeneous.

As previously mentioned, in the resistive state, kinematic V–Av pairs were created at the borders and moved towards the center of the sample. This is another effect of the self-field, since simulations without its consideration, as was also reported in the literature^7^, presented the same voltage step and transition to the resistive state with a vortex street solution; however, the kinematic V–Av pairs presented a behavior opposite to the one described above: the pairs were nucleated at the center of the sample and annihilated at its edges. This change is also linked to the current density modified by the self-field, more specifically, to the changes it produces in the supervelocity.

**Figure 3:**
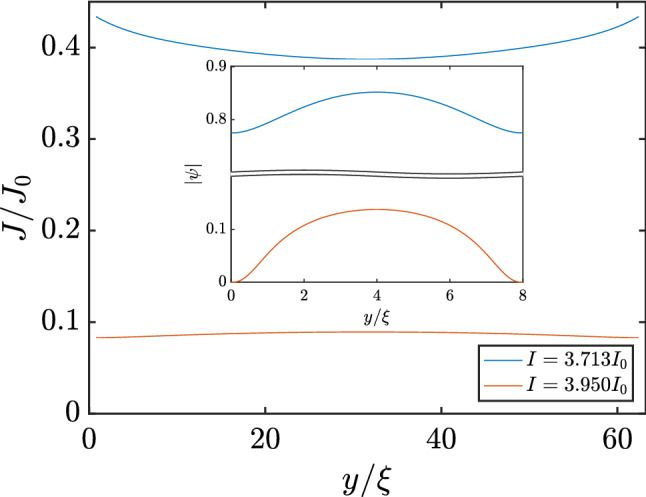
The superconducting density current across the width of the sample, at its center, for two different applied current values, $I=3.713 I_0$ (blue curve) and $I=3.950 I_0$ (red curve). The inset shows the superconducting order parameter across the width of the sample.

The supervelocity, which can be expressed as $\mathbf{v }=\mathbf{J }_s/{\vert \psi \vert }^2$, has its highest value at the point where a vortex nucleates in the sample. For cases without a self-field, the supervelocity had its highest value, for current values right after the first step in voltage, at the center of the sample^7^. On the other hand, when the self-field was properly considered in the simulations, the supervelocity had its highest value at the edges, as shown in Fig. 3. Here the supercurrent density distribution is approximately uniform, but the order parameter is much more suppressed near the borders of the sample, resulting in maximum values for supervelocity in those regions. The currents responsible for the self-field cause the supervelocity to reach maximum values at the edges rather than at its center. This will have an important consequence on the subsequent phenomena encountered.

When increasing the applied current, the system remained at this resistive vortex street solution without significant change until the current reached $I = 6.794 I_0$, where the *I–V* characteristics curve’s slope changed. This caused a peak in the differential resistance, as shown in Fig. 2b. The same phenomenon was observed for numerical simulations that did not consider the self-field effects^7^. However, in that work, this was linked to a change in the positions of the nucleation and annihilation of the kinematic V–Av pairs, which were created at the borders of the sample and annihilated at its center. In the self-field simulations, the creation and annihilation process always took place in the latter form. This raises the question of what is really responsible for the slope change in the *I–V* curve and the maximum values at the differential resistance.

**Figure 4:**
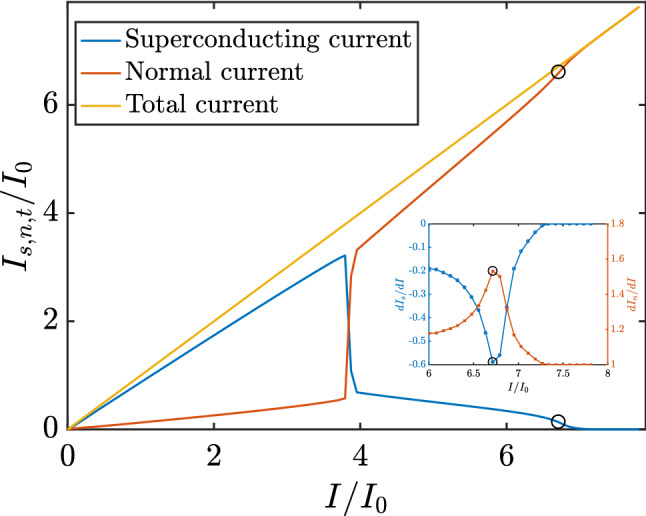
Total superconducting current (blue curve) and total normal current (red curve) that cross through the width of the sample, at its center, as functions of the total applied current. The inset shows the rate of change of the superconducting and normal current as functions of of the applied current.

The differential resistance peak found at $I = I_R = 6.794 I_0$ was accompanied by other interesting phenomena. For instance, there was a decrease in the rate at which the superconducting current was converted to normal current inside the sample. Figure 4 shows the total superconducting ($I_s$) and total normal ($I_n$) currents that passed through the whole width of the sample, at its center, as a function of the total applied current (*I*). For currents below $I = 3.792 I_0$, the total superconducting current increased at a fairly constant rate. However, at the transition point, $I_s$ dropped abruptly while $I_n$ increased substantially. For larger values of the applied current, the superconducting current was converted to normal current at an increasing rate until the applied current reached $I = 6.715 I_0$, just one step $\Delta I$ smaller than $I_R$, where the differential resistance was maximum. At this point, the rate of conversion $dI_n/dI$ reached its maximum value and, thereafter, decreased with increasing applied current. Figure 4 shows the rate of change of $I_s$ and $I_n$ as functions of the applied current *I*. The superconducting current’s rate of destruction reached its maximum at $I = 6.715 I_0$. For greater values, the superconducting current was still being destroyed but at a much lower rate.

**Figure 5:**
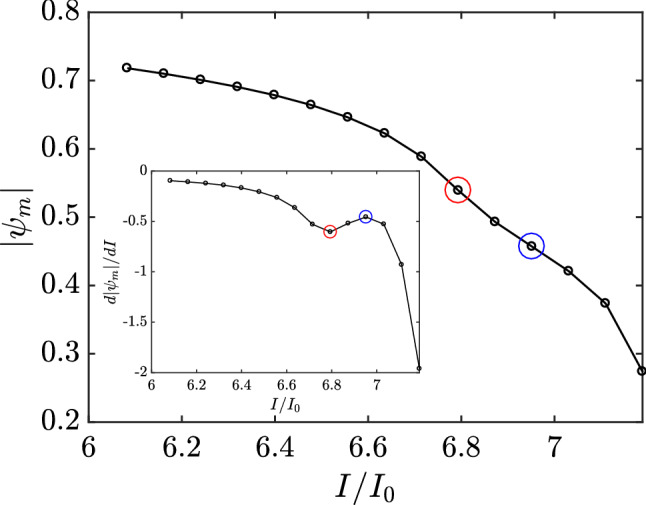
The modulus of the time-averaged superconducting order parameter as a function of the applied current. The inset presents the rate of destruction of the order parameter with increasing current. The point marked with a red circle corresponds to $I = 6.794 I_0$ and the one marked with blue circle to $I = 6.952 I_0$.

Another interesting phenomenon that occurred at $I=I_R$ was the decreased annihilation rate for the superconducting order parameter in the sample. Figure 5 shows the modulus of the time-averaged superconducting order parameter at the center of the stripe as a function of the applied current. The order parameter monotonically dropped to zero as the current approached the value of the superconducting-normal transition, $I = 7.268 I_0$, as is shown in Fig. 2. The inset of Fig. 5 presents the rate of change of the time-averaged superconducting order parameter calculated at the center of the vortex street as a function of the applied current. For currents lower than $I_R$, the order parameter was annihilated at an increasing rate. However, for current values greater than $I_R$, the rate of annihilation decreased until $I = 6.952 I_0$. This was where the superconducting order parameter returned to being increasingly destroyed until the system reached the normal state. These two points are highlighted in Fig. 5.

**Figure 6:**
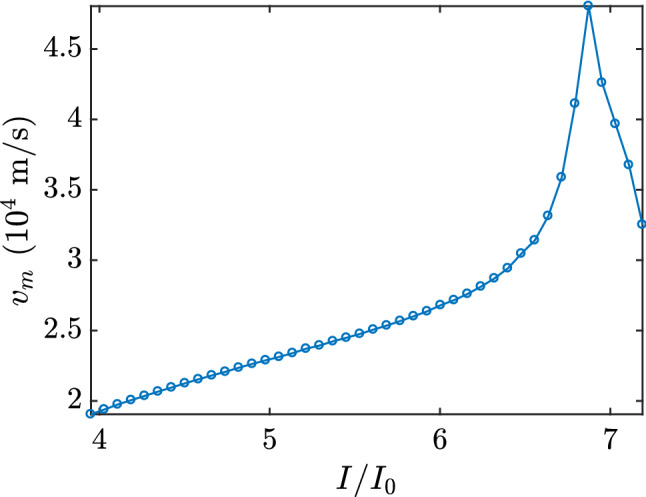
Velocity of the kinematic vortex as a function of the total applied current. The velocity is in real units; we have used $\xi =10$ n$\mu$ and $t_{GL}=6.72$ ps which are typical values for Nb thin films^17^.

In these two processes, the decrease in the rate of conversion of the superconducting current to normal current and the decrease in the rate of annihilation of the superconducting order parameter in the sample can be explained by another phenomenon that took place at $I = 6.873 I_0$, just one step $\Delta I$ above the $I=I_R$. Near this point, the velocity of the kinematic V–Av pairs reached its maximum. Figure 6 shows the average velocity of the kinematic vortex as a function of applied current. The average vortex velocity presented, for currents lower than $I = 6.873 I_0$, a monotonically increasing pattern for increasing applied current, with yet a larger rate of increase for currents near this value. However, this tendency to increase abruptly ceased when the applied current reaches $I = 6.873 I_0$, where the kinematic vortex velocity was maximum. For higher values, the average velocity began to decrease with increasing applied current.

The quasiparticle spectrum changes from superconducting to normal current when a vortex travels across the sample. Thus, with a higher vortex velocity, the quasiparticles switch more rapidly, causing an increase in the rate that superconducting current is being conversed to normal current and an increase in the annihilation rate of the superconducting order parameter. On the order hand, for applied currents larger than $I = 6.873 I_0$, the vortex velocity starts to decrease, consequently causing a reduction in both the conversion rate and annihilation rate.

Furthermore, we have also encountered that the self-field influences the magnitude of the kinematic vortex velocity in the numerical simulations. The average velocity of the kinematic vortex inside the sample remains finite for all values of the applied current, as seen in Fig. 6. This is unlike the average velocity obtained in simulations with the absence of the self-field^7^, which diverge to infinity at $I=I_R$.

To summarize, in this manuscript, we have numerically solved the generalized time-dependent Ginzbug–Landau equation equation and investigated the resistive state for a superconducting stripe driven by an applied transport current. Contrary to previous literature, the calculations have explicitly considered the magnetic self-field induced by the internal currents. We found that the self-field influences the density of the superconducting current by changing the location of the creation and annihilation of the kinematic V–Av pair. The self-field can also alter the type of resistive state found for a given set of geometrical parameters. For instance, a system with a normal contact equivalent to the stripe width, with $\kappa =5.0$ and $\gamma =20$, changes from a PSL resistive state to a vortex street solution when the self-field is included in the simulations. In addition, we also investigated the influence of the kinematic vortex velocity in the resistive state. The results maintained that, above certain applied current values, the vortex velocity ceases to increase and begins to decrease, subsequently decreasing the rate at which that the superconducting current is converted to normal current and decreasing the annihilation rate of the superconducting order parameter. Finally, our results show that the self-field has important consequences to the dynamics of the resistive state. Thus, it cannot be disregarded in similar numerical simulations.

## Methods

We have used the generalized time-dependent Ginzburg–Landau equation (see Refs.^18,19^). In dimensionless form, this equation can be written as

1 $$\begin{aligned} \frac{u}{\sqrt{1+\gamma ^2|\psi |^2}} \left( \frac{\partial }{\partial t} +\frac{\gamma ^2}{2}\frac{\partial |\psi |^2}{\partial t}+i\varphi \right) \psi&\nonumber \\ = -\left( -i\varvec{\nabla }-\mathbf{A} \right) ^2\psi +\psi (1-|\psi |^2)\;.&\end{aligned}$$

The vector potential was determined using the Ampère-Maxwell equation

2 $$\begin{aligned} \frac{\partial \mathbf{A}}{\partial t}+\varvec{\nabla }\varphi = \mathbf{J}_s-\kappa ^2\varvec{\nabla }\times \mathbf{h}\;. \end{aligned}$$

where the superconducting current density is

3 $$\begin{aligned} \mathbf{J}_s = {\mathrm{Re}}\left[ {\bar{\psi }}\left( -i\varvec{\nabla }-\mathbf{A} \right) \psi \right] \;, \end{aligned}$$

and the local magnetic field is related to the vector potential through the equation $\mathbf{h }=\varvec{\nabla }\times \mathbf{A }$.

The equation for scalar potential can be derived from the continuity equation

4 $$\begin{aligned} \frac{\partial \rho }{\partial t} +\varvec{\nabla }\cdot \mathbf{J }=0\;, \end{aligned}$$

where $$\mathbf{J }=\mathbf{J }_s+\mathbf{J }_n$$, and

5 $$\begin{aligned} \mathbf{J }_n=-\left( \frac{\partial \mathbf{A}}{\partial t}+\varvec{\nabla }\varphi \right) \end{aligned}$$

is the normal current density. Supposing that there is no accumulation of charge, we can write $\frac{\partial \rho }{\partial t}=0$, which yields $\varvec{\nabla }\cdot \mathbf{J }=0$. Then, by assuming the Coulomb gauge $\varvec{\nabla }\cdot \mathbf{A}=0$, from (2) we can easily obtain

6 $$\begin{aligned} \nabla ^2\varphi = \varvec{\nabla } \cdot \mathbf{J}_s\;. \end{aligned}$$

Here, lengths are in units of the coherence length, $\xi$, temperature, *T*, is in units of $T_c$, and time is in units of the GL time characteristic $\tau _{GL}=\pi \hbar /8k_BT_c\epsilon u$, where $\epsilon =(T_c-T)/T_c$. In addition, the magnetic field is in units of the upper critical field, $H_{c2}$, the electrostatic potential is in units of $\varphi _0=\hbar /2e\tau _{GL}$, the vector potential is in units of $H_{c2}\xi$, the current density is in units of $J_0=c\sigma \hbar /2e\xi \tau _{GL}$ (where $\sigma$ is the electrical conductivity in the normal state), and the order parameter is in units of $\psi _0 = \sqrt{|\alpha |/\beta }$ (the order parameter in the Meissner state). Lastly, $\alpha$ and $\beta$ are the GL phenomenological constants, and $\kappa =\lambda /\xi$ is the Ginzburg–Landau parameter, and $\lambda$ is London penetration length. The constant $u=5.79$ was derived from the first principles in Refs. ^18,19^.

We have solved Eqs. (1), (2), and (6) numerically for the geometry exhibited in Fig. 1a. Along all sides of the film, $\mathbf{n }\cdot \varvec{\nabla }\varphi =0$ except on the normal contacts where $\mathbf{n }\cdot \varvec{\nabla }\varphi =-J_a$. In the limit of thickness $d\ll \xi$ inside the film, we may consider that the self-field was nearly perpendicular to the film along the *z* direction. The validity of this approximation has been rigorously proved in Ref. ^20^ to be good for large $\kappa$, typically $\kappa \gtrsim 5$^21^. Thus, within this approximation, the boundary conditions for the self-field are

7 $$\begin{aligned} h_z\bigg (x,\pm \frac{w}{2}\bigg )= & {} \pm \frac{J_aa}{2\kappa ^2}\;, \nonumber \\ h_z\bigg (\pm \frac{L}{2},y\bigg )= & {} {\left\{ \begin{array}{ll} \dfrac{J_aa}{2\kappa ^2}\;, &{} \dfrac{a}{2} \le y \le \dfrac{w}{2}\;, \\ \dfrac{J_ay}{\kappa ^2}\;, &{} -\dfrac{a}{2} \le y \le \dfrac{a}{2}\;, \\ -\dfrac{J_aa}{2\kappa ^2}\;, &{} -\dfrac{w}{2} \le y \le -\dfrac{a}{2}\;, \end{array}\right. } \end{aligned}$$

which can be easily obtained from the Ampère’s law $\oint \,\mathbf{h }\cdot d\mathbf{l }=Id/\kappa ^2$ in dimensionless units; here $I=\int J_x(y)\, dy$. Since the thickness of the film is very small and homogeneous, then $h_z$ does not depend on *d*. This same assumption has been used in, for instance, Ref. ^15^. The order parameter is determined at the border of the sample using the Neumman boundary condition $\mathbf{n }\cdot \left( -i\varvec{\nabla } -\mathbf{A} \right) \psi =0$ which assures that the perpendicular component of the superconducting current density vanishes at all sides of the sample.

We solved the equations upon using the link-variable method as sketched in Ref.^22^. The equations were discretized in a mesh-grid of size $\Delta x=\Delta y=0.1\xi .$

As a final remark on our method, we emphasize that our approximation depends on the smallness of the $h_y$ component of the magnetic field in our sample. In the supplementary material, we argue that, for the geometry under investigation, this indeed occurs.

## Supplementary information

Supplementary Information.

## Data Availability

The code used to carry out the numerical simulations can be obtained freely by request to the corresponding author.
