# Supplementary material for: Ultra-fast kinematic vortices in mesoscopic superconductors: the effect of the self-field
Source: Sci Rep. 2020 Oct 29;10:18662. doi: 10.1038/s41598-020-75748-5 (PMC7596518; doi:10.1038/s41598-020-75748-5)
Supplement: Supplementary file 1 — Supplementary Information. [file 41598_2020_75748_MOESM1_ESM.pdf]

Supplementary Material to Ultra-fast Kinematic Vortices in Mesoscopic Superconductors: The Effect of the Self-Field

Leonardo Rodrigues Cadorim[1], Alexssandre de Oliveira Junior[2], and Edson Sardella[1,*]

[1]Departamento de Física, Faculdade de Ciências, Universidade Estadual Paulista (UNESP), Caixa Postal 473, 17033-360, Bauru-SP, Brazil
[2]Instituto de Física Gleb Wataghin, Universidade Estadual de Campinas, P.O. Box 6165, CEP 13083-970, Campinas, Sao Paulo, Brazil
[*]edson.sardella@unesp.br

May 1, 2020

Justification for neglecting h_y

In this Section we will show that, in the limit of a very thin film, the $h_y(x, y)$ component of the self-field is negligible in comparison to the $h_z(x, y)$ component. We start by considering the Biot-Savart law, which in reduced units is given by:

$$\mathbf{h}(\mathbf{r}) = \frac{1}{4\pi\kappa^2}\int \frac{\mathbf{J}(\mathbf{r}') \times (\mathbf{r}-\mathbf{r}')}{|\mathbf{r}-\mathbf{r}'|^3} d^3r' \; . \tag{1}$$

For simplicity, let us consider a film carrying an uniform current $\mathbf{J} = J_a\hat{\mathbf{x}}$. We have:

$$\mathbf{J}(\mathbf{r}') \times (\mathbf{r}-\mathbf{r}') = J_a(y-y')\hat{\mathbf{z}} - J_a(z-z')\hat{\mathbf{y}} \; . \tag{2}$$

Substituting Eq. (2) into Eq. (1), we obtain:

$$\mathbf{h}(\mathbf{r}) = \frac{J_a}{4\pi\kappa^2}\int_{-d/2}^{d/2}\int_{-w/2}^{w/2}\int_{-\infty}^{\infty} dz'dy'dx' \left[\hat{\mathbf{z}}\frac{(y-y')}{R^3} - \hat{\mathbf{y}}\frac{(z-z')}{R^3}\right] , \tag{3}$$

where $R = \left[(x-x')^2 + (y-y')^2 + (z-z')^2\right]^{1/2}$. Note that, though our stripe is finite, we can extend the x' derivative to infinite due to the fact that we can assume, for theoretical proposes, the normal contact as long as is convenient.

Performing the integral over x', we find:

$$\mathbf{h}(\mathbf{r}) = \frac{J_a}{4\pi\kappa^2}\int_{-d/2}^{d/2}\int_{-w/2}^{w/2} dz'dy' \left\{\hat{\mathbf{z}}\frac{(x'-x)(y-y')}{\left[(y-y')^2 + (z-z')^2\right]R} - \hat{\mathbf{y}}\frac{(x'-x)(z-z')}{\left[(y-y')^2 + (z-z')^2\right]R}\right\}\Bigg|_{-\infty}^{\infty} \tag{4}$$

from which we obtain:

$$\mathbf{h}(\mathbf{r}) = \frac{J_a}{4\pi\kappa^2}\int_{-d/2}^{d/2}\int_{-w/2}^{w/2} dz'dy' \left\{ \hat{\mathbf{z}}\frac{2(y-y')}{\left[(y-y')^2+(z-z')^2\right]} - \hat{\mathbf{y}}\frac{2(z-z')}{\left[(y-y')^2+(z-z')^2\right]} \right\} . \quad (5)$$

Integrating the z component of the field with respect to y' and the y component with respect to z', one can easily find:

$$h_z(\mathbf{r}) = -\frac{J_a}{4\pi\kappa^2}\int_{-d/2}^{d/2} dz' \ln\left[\frac{(y-w/2)^2+(z-z')^2}{(y+w/2)^2+(z-z')^2}\right] , \quad (6)$$

and

$$h_y(\mathbf{r}) = \frac{J_a}{4\pi\kappa^2}\int_{-w/2}^{w/2} dy' \ln\left[\frac{(y-y')^2+(z-d/2)^2}{(y-y')^2+(z+d/2)^2}\right] . \quad (7)$$

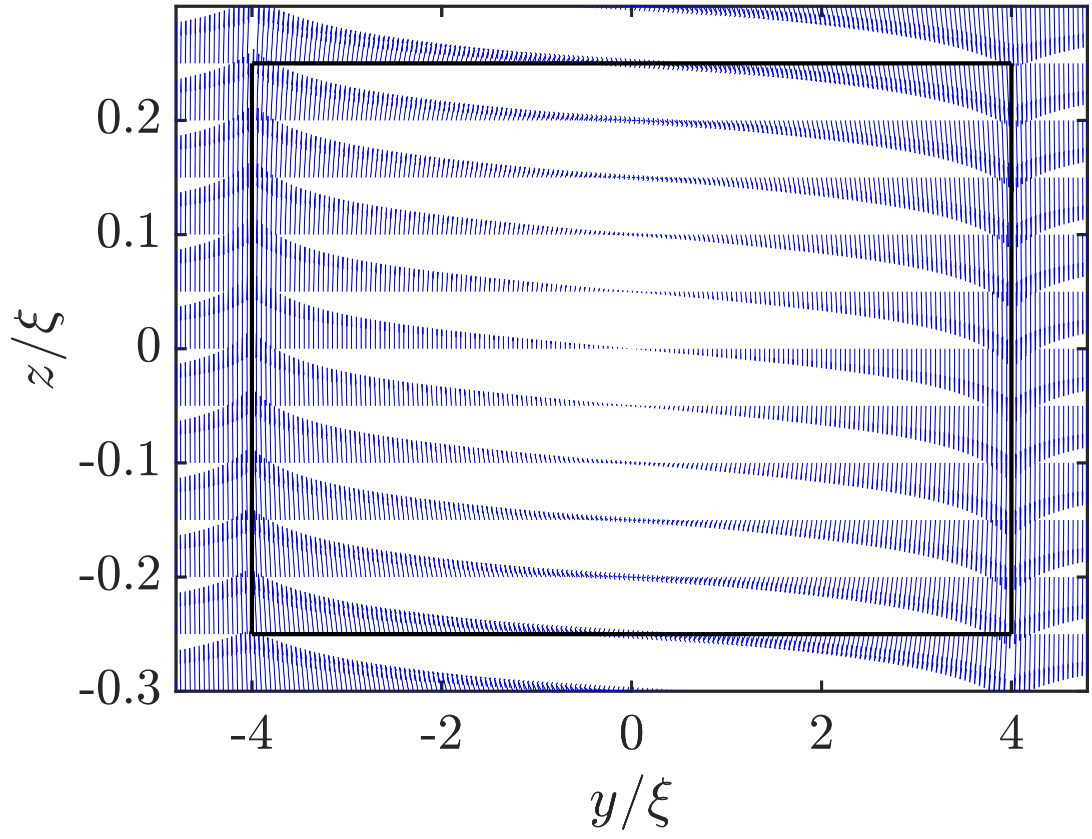

Figure 1: Vector field for $L = 12\xi$, $w = 8\xi$, $d = 0.5\xi$ and normal contact width $a = 8\xi$. The darker lines indicate the borders of the film.

These two integrals can be easily obtained. We have:

$$h_z(\mathbf{r}) = \frac{J_a}{4\pi\kappa^2}\Bigg[(z-d/2)\ln\left[\frac{(y+w/2)^2+(z-d/2)^2}{(y-w/2)^2+(z-d/2)^2}\right] - (z+d/2)\ln\left[\frac{(y+w/2)^2+(z+d/2)^2}{(y-w/2)^2+(z+d/2)^2}\right] - \quad (8)$$

$$-2(y-w/2)\arctan\left(\frac{(z-d/2)}{(y-w/2)}\right) + 2(y-w/2)\arctan\left(\frac{(z+d/2)}{(y-w/2)}\right) + \quad (9)$$

$$+2(y+w/2)\arctan\left(\frac{(z-d/2)}{(y+w/2)}\right) - 2(y+w/2)\arctan\left(\frac{(z+d/2)}{(y+w/2)}\right)\Bigg], \quad (10)$$

and

$$h_y(\mathbf{r}) = \frac{J_a}{4\pi\kappa^2}\Bigg[-(y-w/2)\ln\left[\frac{(y-w/2)^2+(z+d/2)^2}{(y-w/2)^2+(z-d/2)^2}\right]+(y+w/2)\ln\left[\frac{(y+w/2)^2+(z+d/2)^2}{(y+w/2)^2+(z-d/2)^2}\right]+ \tag{11}$$

$$+2(z-d/2)\arctan\left(\frac{(y-w/2)}{(z-d/2)}\right)-2(z-d/2)\arctan\left(\frac{(y+w/2)}{(z-d/2)}\right)- \tag{12}$$

$$-2(z+d/2)\arctan\left(\frac{(y-w/2)}{(z+d/2)}\right)+2(z+d/2)\arctan\left(\frac{(y+w/2)}{(z+d/2)}\right)\Bigg]. \tag{13}$$

The vector field of this magnetic field is shown in Figure 1. As can be seen, the magnetic field lines are approximately perpendicular in almost all the sample, as assumed in the manuscript. The only region of the sample where the field lines are not perpendicular is close to the film surface ($|z| \approx d/2$) and in the vicinity of $y = 0$. In this regions, though, we note that the magnitude of the magnetic field is much smaller than at the borders, where h_z is predominant. This analysis supports our approximation to take $h_y = 0$ throughout the sample and consider the magnetic field lines as straight lines along the z direction, as depicted in Figure 1 of our manuscript.

Further, it can be shown that, our approximation is able to capture the important effects of the self-field to the dynamics of the kinematic vortex This goes as follows. The self-field contributes to the physics of the studied problem in two different manners. First, the self-field influences the interaction of the kinematic V-Av pair, consequently influencing the velocity curve as a function of applied current as presented in our manuscript. Here, we resort to our previous argument, that h_y is only appreciable in regions where the total field is small, being completely negligible in the regions where the field matters most. Thus, this small contribution of h_y to the V-Av interaction could certainly not lead to any qualitative change in our results, *i.e.*, it can only slightly increase or decrease the already existing V-Av interaction, leading, at most, to small quantitative changes in our results.

Second, the self-field influences the current distribution along the width of the sample. This distribution is important because, as is well known and is stated in our manuscript, the point at which a vortex (anti-vortex) penetrates into the sample depends on where the current distribution is maximum. In this point, h_y plays absolutely no role in the distribution of the current along the width of the sample, once a field in the y direction gives no force component in this same direction, thus leaving the given current distribution unchanged. The fact that the location of the creation of the kinematic V-Av pair is one of the main results of our work, once more proves that the approximation used is indeed capturing the most important aspects of the self-field for the dynamics of the kinematic vortex. The two arguments given above show that, contrary to what is claimed by the reviewer, our approximation is suitable for the problem under investigation and is capable of capturing its underlying physics.
